# Cyberharassment Victimization on Three Continents: An Integrative Approach

**DOI:** 10.3390/ijerph191912138

**Published:** 2022-09-25

**Authors:** Marko Mikkola, Noora Ellonen, Markus Kaakinen, Iina Savolainen, Anu Sirola, Izabela Zych, Hye-Jin Paek, Atte Oksanen

**Affiliations:** 1 Faculty of Social Sciences, Tampere University, 33100 Tampere, Finland; 2 Institute of Criminology and Legal Policy, University of Helsinki, 00100 Helsinki, Finland; 3 Department of Social Sciences and Philosophy, University of Jyväskylä, 40014 Jyväskylä, Finland; 4 Department of Psychology, University of Córdoba, 14071 Cordoba, Spain; 5 Department of Advertising & Public Relations, Hanyang University, Ansan 15588, Korea

**Keywords:** cyberharassment, victimization, routine activity theory, general theory of crime, sense of mastery

## Abstract

This article introduces and applies an integrative model of cyberharassment victimization. The model combines routine activity theory (RAT), the general theory of crime (GTC), and the personal resources approach to analyze risk factors for victimization while acknowledging the protective role of a sense of mastery. Survey respondents were aged 15 to 25 years (*N* = 4816) from the U.S., Finland, Spain, and South Korea. Logistic regression models were used to analyze cyberharassment victimization. RAT-related factors were positively associated with cyberharassment victimization. Low self-control was positively associated with cyberharassment victimization in the U.S., Finland, and Spain but not in South Korea. The sense of mastery was negatively associated with cyberharassment victimization in the U.S., Finland, and South Korea but not in Spain. Protective factors against cyberharassment victimization should be utilized in future studies as adequate knowledge of protective factors could assist policymakers in generating preventative measures against cyberharassment. Our study demonstrates the benefits of integrating criminological theories and protective factors in studies using cross-national data to gain a better understanding of the dynamics of cyberharassment.

## 1. Introduction

Cyberharassment and cybercrime victimization are prevalent negative consequences of Internet use, impacting numerous individuals around the globe [1,2,3,4,5,6]. The growing threat of cybervictimization is an everyday concern especially for younger people who live in technologically advanced countries [3]. Experiencing cyberharassment (i.e., online harassment) among adolescents and young adults has been associated, for instance, with difficulties at school, experiences of sexual abuse, and delinquent behavior [2,4,7,8,9].

A considerable number of prior studies have utilized routine activity theory [1,4,5,10,11,12,13,14] or self-control from the general theory of crime (GTC) [15,16] to explain the situational or personal factors behind online victimization. In addition, some of the previous victimization studies have combined RAT and the general theory of crime to investigate both offline and online contexts of victimization [17,18,19,20,21].

The need to integrate established theories of crime has arisen from the criticism of the mentioned theories’ suitability to singularly explain online victimization while Internet users globally are facing new types of online crimes [5,22,23,24,25,26]. A notable shortcoming of integration of theories, however, is that the approach fails to recognize the role of personal resources in avoiding victimization experiences. Earlier studies have shown that the risk of online victimization is explained both by situational risk factors (i.e., risky online environments) and by individuals’ behavior in risky situations [26]. This means that individuals differ in their adaptive coping skills that may help them to avoid victimization experiences online. Thus, there is a need for criminological research that adds the perspective of personal resources to the situational and personal risk factors (RAT and GTC).

In this paper, the objective is to combine situational (RAT) and personal (self-control; GTC) risk factors and the protective resources perspective (sense of mastery) in predicting online harassment victimization. Using multivariate methods, we analyze how RAT and impulsivity as a key dimension of self-control, and sense of mastery are related to online harassment, and which of the approaches predicts the risk most strongly [27]. This kind of explanatory power analysis has not been performed before. As cyberharassment is a global phenomenon it is crucial to use cross-national data to determine if same risk factors can explain cyberharassment in different cultural settings and societies in a similar manner [2,3,6]. Thus, we analyze multinational data from Finland, South Korea, Spain, and the U.S. These four countries from three different continents were chosen as adolescents and young adults in these technologically advanced countries are active Internet and social media users [28,29]. Previous studies have shown that country differences exist in the prevalence of online harassment victimization [4,14].

Our approach enables the analysis of situational and personal risk factors and personal protective resources for the risk of cybercrime victimization. Specifically, we analyzed how routine activities, impulsivity, and mastery are associated with adolescents’ and young adults’ cybervictimization by combining RAT, GTC, and sense of mastery into an integrative model of cybervictimization. Our study contributes a novel approach to the investigation of cybercrime victimization and its associated factors.

### 1.1. Cyberharassment

Beran and Li defined cyberharassment as “a new method for an old behavior” [7]. This means that the old behavior, a physical encounter between an offender and a victim, has a new manifestation known as cyberharassment, in which harassment occurs through the use of a new mediator, namely, information technology. In more recent literature, cyberharassment is defined as intentional and repetitive aggression perpetrated by individuals and groups using different types of electronic apparatus against targets who cannot easily protect or defend themselves [30,31]. Cyberharassment can be direct, in which case the offensive material is sent directly to the receiver, or in-direct, in which case the offensive material is posted online for others to find [14,32]. Further, whereas face-to-face harassment consists of intentionality, repetition, and power imbalance, cyberharassment can occur without these elements [23,31,33,34].

Cyberharassment can occur in all life stages, from childhood to adulthood, and cause negative outcomes such as depression, delinquency, stress, and substance use [13,14,32,35,36]. Existing studies have shown that certain types of behavior or Internet usage increase the risk of being victimized online in general. According to a study by Näsi and colleagues, visiting websites relating to eating disorders was positively associated with an increased risk of harassment victimization [14]. Similar findings on online routines and cybervictimization have also been reported in other studies [37]. Compulsive Internet use, low self-control, and the use of different social media services have also been related to increased risk of victimization [15,38]. Additionally, age, sex, socio-economic status, personality, self-esteem, loneliness, and impulsivity have been found to be associated with cyberharassment victimization. It is also vital to notice that cyberharassment on the Internet and social media services is an ever-evolving phenomenon. Thus, the factors behind cyberharassment victimization may also change over the time [39,40,41,42]. 

### 1.2. Existing Studies Utilizing RAT and CTG

A considerable number of prior studies have utilized RAT and its three core components: exposure to motivated offender, target suitability, and guardianship to explain factors behind cybervictimization [1,4,5,11,12,13,14]. Vakhitova and colleagues concluded that victims’ online routine activities are significant factors behind cybervictimization [5]. In addition, according to earlier studies, the risk of online harassment victimization increases when adolescents and young adults use social networking sites, associate with peers who harass others online or post sensitive personal information online [10,11,22,42]. To summarize, especially exposure to motivated offender and target suitability are key components for a crime to occur online [4,5,12,13,43].

According to Navarro and Jasinski, the third core component, capable guardian, has turned out to be a problematic component to study [43]. Capable guardian can be an authority or technical solution such as a web content filtering solution, but also anyone whose mere presence can prevent a crime from happening [22,44,45,46]. The lack or absence of guardianship can increase the possibility of crime or harassment to occur. Existing studies suggest that certain types of individual maladjustments such as loneliness can lead to difficulties in peer relationship, e.g., social withdrawal, submissiveness, or lack of social support [47,48,49]. Thus, loneliness can lead to a situation where an individual has no one to share their problems with or friends who could assist in times of trouble, i.e., lack or absence of guardianship, which in turn can increase the likelihood of victimization. [22,42,50,51].

Self-control from the general theory of crime (GTC) has been used somewhat successfully with RAT to explain victimization in the physical (offline) or digital (online) context, using mostly national data [17,18,19,20,21,52]. Overall, the number of multinational studies on cyberharassment victimization using RAT, CTG, or both is relatively low, with some exceptions [4,14,18,42]. Mikkola with his team found that RAT-related factors together with impulsivity from GTC, predicted higher possibility of cybervictimization. Specifically, RAT factors pertaining to social media use, sharing information online, sending offensive messages to others, and loneliness, and impulsivity from GTC were associated with a higher risk of cybervictimization [42]. Näsi with his team found that the only significant variable from RAT was exposure to offender [4]. In addition, these studies focused purely on risk factors. Only few studies have combined risk factors with possible protective factors [53].

The strength of the theory integration approach is that it combines two established criminological theories: the situational perspective of RAT with the personal risk factors delineated by GTC. Together, these theories can provide a stronger framework to explain online victimization. However, Ngo and Paternoster stated that other theoretical frameworks should also be applied to better explain cybervictimization as the results vary based on type of cybercrime victimization. Thus, in the current study we are using an integrated model that includes protective factors and utilizes multination data to better explain cyberharassment victimization [17].

### 1.3. Routine Activity Theory

Routine activity theory (RAT) is one of the most common theories used to explain offline and online crime and victimization [45]. RAT is a situational and opportunistic theory according to which crime most likely occurs when a motivated offender and a suitable target meet in a situation lacking a capable guardian. Most commonly such a situation occurs because of the routine lifestyles of the target, who because of daily routines, becomes visible and thus, an attractive target to the offender. Consequently, crime is not a random occurrence as it follows the routine patterns of people’s everyday social life [12]. According to RAT, there are always motivated offenders who are constantly seeking suitable targets both online and offline [45,54].

RAT was initially developed to explain crime in the physical (i.e., offline) world when an offender and a target meet at the same time in a physical situation and place that lacks a capable guardian. According to Marcus Felson, cybercrime (i.e., online crime) follows the same principles as crime in the physical world even though the offender, victim, and the guardian do not necessarily coexist in the same physical space and time [54]. Instead, offenders can send direct threats or leave a threat for the victims to find the next time they use their e-mail or other means of communication on the Internet.

The transition of the theory from the physical to virtual world has created criticism in terms of RAT’s capability to explain online victimization [22,55]. Regardless of the criticism, RAT has been used to explain online victimization in a wide range of cybervictimization studies over the past decades [4,11,13,56,57,58,59,60]. Some earlier studies have also identified limitations for using RAT and its strictly defined core components to explain online victimization. The criticism has been directed to the fact that a target and an offender do not necessarily converge within a shared time and space and to the definition or the role of the capable guardian. These earlier studies have also shown that not all three core components of RAT are equally significant [22].

### 1.4. Impulsivity in General Theory of Crime

Extensive social psychological and criminological research has established that self-control and the ability to regulate it impact individuals’ quality of life. Individuals with high self-control are successfully managing their lives and face fewer problematic life situations than individuals with low self-control. Individuals with high self-control are more successful in controlling their behavior and seeing the long-term consequences of their own choices and actions [61,62].

Impulsivity is one of the six key elements in the GTC introduced by Michael Gottfredson and Travis Hirschi [52]. The other five dimensions are: preference for simple tasks, risk-seeking, physicality, self-centeredness, and bad temper. According to the GTC, individuals with low self-control are more likely to commit crimes or deviant acts than those with high self-control. Thus, high self-control acts as a preventing element between the actor and the crime. However, low self-control is found to increase both the risk of offline and online victimization [56,63,64,65].

Even though self-control as a construct cannot be reduced to impulsivity, it can be considered a key sub-dimension of self-control, as low self-control often manifests as impulsive behavior [27,66,67]. Impulsive individuals often look for immediate gratification without considering the long-term effects of their actions. Impulsivity has been found to relate to certain risky routines, such as delinquent or aggressive behavior. This, in turn, can expose individuals with low self-control to motivated offenders, increasing their risk of becoming suitable targets and becoming victimized, especially in situations lacking guardianship [19,45].

Furthermore, in existing studies, low self-control has been successfully used to explain an increased risk of victimization [15,16,63,64,65,68]. According to previous studies, people with low self-control tend to make impulsive decisions, increasing their exposure to offenders [68]. However, these studies—as well as studies using indicators derived from RAT in explaining online victimization—have limitations related to sample issues. Most of these studies are based on college student samples, limiting their findings’ generalizability [1,10,13]. In addition, the studies are based mostly on data from one country although there are studies suggesting differences between countries in explaining online victimization [1,4,10,13,14].

To advance the knowledge of online victimization, more cross-national analyses are needed. In addition, more factors potentially associated with online victimization need to be introduced to the studies to gain further knowledge of risk as well as protective factors. In this research, we use factors derived from both RAT and the theory of self-control as risk factors for online victimization, and we introduce a sense of mastery as a potentially protective factor against cybervictimization.

### 1.5. Sense of Mastery

Cyberharassment can cause negative outcomes such as depression, delinquency, stress, and substance use [32,35]. Thus, there is constant need to develop further harassment prevention programs. However, positive attributes, such as optimism, positive mental health, morality, empathy, and self-control have been seen as protective factors against negative outcomes of cyberharassment [35,50,69,70,71,72,73]. Majority of existing studies using well-established criminological theories are concentrating on factors that increase the risk of victimization. From the victimization prevention point of view, knowledge on protective factors is equally important. Otherwise, prevention plans would be solely based on risk reduction approach [50].

The sense of mastery is one of the psychological resources people invoke to help them withstand difficulties or threats posed by different occurrences in their way of life. These resources that reside within the self can be used as efficient barriers or coping abilities against negative consequences of social strain [74]. In earlier studies, a high sense of mastery was found to predict a lower likelihood of violent victimization in the future [70,75,76,77]. This suggests that a sense of mastery may make individuals more capable of controlling threatening situations or appear less vulnerable to others. Furthermore, adolescents’ mastery continues to develop across the individual’s entire lifespan [70,74,78].

Mastery is not simply the opposite of impulsivity in broader terms. Rather, it is an important positive personal attribute that promotes an individual’s well-being and capability to cope with changes in one’s life compared to being ruled fatalistically by others [62]. Impulsivity, on the other hand, is seen as an attribute leading to troublesome situations. Therefore, in this study, we treat impulsivity as a negative attribute, suggesting that high impulsivity predicts higher odds of getting victimized. Whereas an impulsive personality is related to thrill-seeking behavior and getting immediate gratification without consideration of future consequences, the sense of mastery is found to be a protecting resource that can facilitate effective coping strategies [74,79,80].

### 1.6. Current Study

The theoretical frameworks of RAT and GTC have been utilized in earlier studies to explain both offline and online cybervictimization [18,19,20,21]. In this study, we investigate how integrating RAT with the GTC works together with the sense of mastery in explaining cyberharassment among adolescents and young adults. The integrative approach also allows us to compare how these different predictors perform in explaining online harassment. We test our hypotheses using samples from four different countries: the United States, Finland, Spain, and South Korea.

Existing studies have shown how exposure to motivated offenders can lead to cybervictimization, especially when visiting potentially risky websites or by being active on the Internet and on social media sites [9,12,13]. However, other recent studies have found that online exposure to offenders does not increase the odds of cybervictimization [81]. The mixed results could be explained by the general change in the Internet and social media over the years. Therefore, we study how the use of potentially harmful websites and sharing content on social media affects the visibility of targets to the offenders:

**Hypothesis** **1** **(H1).**  *Visiting potentially harmful websites and sharing content on social media increase targets’ visibility to offenders, thus leading to cybervictimization*.

Meaningful social relationships protect from many risks and harms encountered on the Internet [82]. Regarding cyberharassment victimization, we approach the lack of guardianship via a sense of loneliness, that is, the perceived deficiency and disconnection in one’s relationships [48,83]. For example, a sense of being misunderstood or not being able to talk openly with parents or peers might prevent sharing personal things such as harassment experiences, which in turn might lead to further victimization. It is uncertain, or at least difficult, to determine how capable guardianship functions within the social media space [22,84]. We think guardianship can refer to very different social agents, from onlookers to accompanying friends. However, given that online and offline social networks tend to overlap, we assume that inclusion in social networks serves as a source of guardianship on social media as well [85]. Similarly, exclusion and lack of social relationships means the absence of this protection on social media interaction as well.

**Hypothesis** **2** **(H2).**  *Loneliness is associated with cyberharassment victimization.*

Existing studies have demonstrated a link between cybervictimization and low self-control [15,16,56,63,64,65]. Based on the idea of impulsivity being a key subdivision of self-control, we expected high impulsivity to be a significant factor explaining cybervictimization [27,66].

**Hypothesis** **3** **(H3).**  *Impulsivity is associated with cyberharassment victimization.*

In our integrative approach to cybervictimization, we added the concept of a sense of mastery into the model. Our aim is to analyze the social psychological dynamics of cyberharassment victimization as idealized in positive criminology. In positive criminology, the focus is on individuals’ positive resources, which can act as preventative measures against victimization [72].

**Hypothesis** **4** **(H4).**  *Sense of mastery is a protective factor against cyberharassment victimization.*

## 2. Method

### 2.1. Participants and Procedure

Respondents were aged 15 to 25 in Finland (*N* = 1200, 50.0% female), The United States (*N* = 1212, 50.2% female), South Korea (*N* = 1192, 50.4% female), and Spain (*N* = 1212, 48.8%). Data were collected in April 2017 in Finland, followed by the United States in January 2018 and South Korea in April 2018, and Spain in January 2019.

The research group designed the survey, and it was maintained on the University server. Respondents were drawn from the pool of respondents provided by Dynata (former Survey Sampling International). Dynata combines respondents from different sources to maintain the consistency of samples. They recruit respondents using random digit dialing, banner ads, and other permission-based techniques. In each target nation, Dynata sent out e-mail invitations to respondents who had previously volunteered to participate in research surveys [57,86,87].

Our data collection was set to mirror the population of the 15 to 25 age group in each country. In order to achieve data that mirrors current populations as closely as possible, quotas were set for age and sex especially. Data collection was achieved by using a 15 min web-based survey that included validated measures on Internet use and excessive behaviors among young people [88].

The original survey was designed in Finnish and was then translated into English. The translation was carried out only for those measures that had not been translated and validated into English before. In other cases, existing translations of measures and batteries were used. The translation process from Finnish to English went through a back-translation to affirm the survey’s internal consistency and accurate matching of the items. The English version was then translated into Korean by proficient Korean and English speakers. Lastly, the English version was translated into Spanish by proficient Spanish and English speakers. In addition, both the South Korean and Spanish versions of the survey went through the final careful back-translation process to ensure both internal consistency and accurate matching of the items.

The research procedure was reviewed by the Ethics Committee of the Tampere region in Finland before implementation. Their statement (Decision 62/2016) concluded that the research did not involve any ethical issues.

The dependent variable, cyberharassment victimization, was measured using the question, “In your own opinion, have you ever been a target of online harassment; for example, have people spread private or unfounded information about you or shared pictures of you without your permission?” with yes or no response choices. The self-report approach to cybervictimization research has been utilized in existing studies and has yielded meaningful results [56,58,82,89].

All independent variables showed acceptable interitem reliability (Cronbach’s alpha). Descriptive statistics of independent variables, means, standard deviations, and reliability coefficients are included in Table 1.

### 2.2. Routine Activity Measures

Three items were measured for routine activity: visiting potentially harmful websites, sharing content on social media, and loneliness. The variable danger sites was created by using the question, “How often do you use the following online sites and services?” The following answer choices were provided: “Dark web (for example, Tor, Freenet, I2P),” “Online casino sites or other sites by gambling companies,” and “Online gambling forums or gambling communities.” Each option had a response scale from 0 (never) to 3 (daily). On the final scale, danger sites ranged from 0 to 9. Variable social media sharing was created using the questions, “How often do you share content on social media?” and “How often do you upload pictures of yourself into social media?” The response scale was from 1 (less than once a year) to 7 (daily). Both variables were summarized, and higher scores indicated higher time spent on dangerous sites and social media use. Both variables were standardized for the multivariate models.

The absence of guardianship is treated as a lack of meaningful social relationships that is manifested in feelings of loneliness. Loneliness and lack of meaningful relationships are important factors in predicting an individual’s psychological health and well-being [61]. Social media services function as a source of fulfilling individuals’ social needs, such as avoiding loneliness [90,91]. Social media services can also provide individuals an opportunity to reduce loneliness and the discrepancy between desired and existing meaningful social relationships [83]. Therefore, lonely people tend to develop compulsive Internet use behavior models, which in turn can lead to negative outcomes in their life. Thus, loneliness has been related to online and offline victimization [92,93]. The variable loneliness was measured with the 3-item short loneliness scale based on the Revised UCLA Loneliness Scale (R-UCLA), which is used as a unidimensional measure of loneliness [48]. The scale has been widely used in multinational loneliness studies [94,95]. The response scale ranged from 1 (*rarely*) to 3 (*often*). All items were summed. A higher score indicates a higher sense of loneliness. The variable was standardized for the multivariate models.

### 2.3. Self-Control and a Sense of Mastery Measures

Impulsivity was measured using the Eysenck Impulsiveness Scale (EIS) [96]. EIS is composed of five items. Each item offers a statement regarding one’s behavior and inquires how the respondent would react on most occasions (e.g., “Do you often get into trouble because you do things without thinking?”). Answer choices are binary 1 (yes) and 0 (no). A sum composite was created, and the final scale ranged from 0 to 5, with higher scores indicating a higher level of impulsivity. One item, “Do you usually think carefully before doing anything?” was reverse-coded. All items were summed, and the variable was standardized.

The sense of mastery was measured using the 7-item Pearlin Mastery Scale [74]. The scale consists of seven questions measuring the extent to which an individual regards the events of their life as under their personal control compared to being fatalistically ruled. The response scale ranged from 1 (strongly agree) to 5 (strongly disagree), with higher scores indicating a higher sense of mastery. Two of the items, “What happens to me in the future mostly depends on me” and “I can do just about anything I really set my mind to,” were reverse-coded. All items were summed, and the variable was standardized.

### 2.4. Control Variables

Variables age and sex were used as control variables. Only respondents aged between 15 and 25 years were accepted into the study. Age was treated as a continuous variable. Sex was measured by asking the participant to indicate whether they were male or female (0 *=* male, 1 *=* female).

### 2.5. Statistical Techniques

We provide descriptive statistics on cyberharassment victimization in Table 1, a zero-order correlation matrix in Table 2, and the main analyses based on logistic regression conducted with Stata 16.1 software (StataCorp LLC, College Station, TX, USA) in Table 3. In Table 3, we report odds ratios (ORs), *p*-values, and average marginal effects (AMEs). Additionally, chi-square (χ²) log-likelihood coefficients are presented for each model with the pseudo coefficients of the determination (Nagelkerke pseudo-R²). All four countries studied were treated separately, and the results are reported per country.

## 3. Results

For all four countries combined (*N* = 4816), a total of 832 (17.3%) respondents aged between 15 and 25 years reported being victims of cyberharassment (see Table 1). Of all cyberharassment victims, 46% were male, and cyberharassment victimization was found within all age groups. Overall, older respondents were more likely than younger participants to be victims of cyberharassment.

On a country level, 23.3% of the U.S. participants, 20.8% of the Finnish participants, 18.3% of the Spanish participants, and 6.5% of the South Korean participants reported being victimized. In all other countries, females were more likely to be cyberharassed than males, except in South Korea, where the number of male victims was higher than that of female victims.

Table 3 reports the results of logistic regression analysis for each country for all independent variables used in the study. In the U.S., age (OR = 1.06, *p* = 0.013) and female sex (OR = 1.60, *p* = 0.002) were positively associated with cyberharassment victimization, while this was not the case in Finland (OR = 0.97, *p* = 0.278 for age, and OR = 0.92, *p* = 0.613 for sex). Female sex in Spain (OR = 1.51, *p* = 0.010), and younger age in South Korea (OR = 0.90, *p* = 0.007), were significantly associated with online harassment victimization.

The RAT-related variables (danger sites, social media sharing, and loneliness) were positively associated with cyberharassment victimization in all four countries. Impulsivity was positively associated with cyberharassment victimization in the U.S. (OR = 1.34, *p* < 0.001), Finland (OR = 1.19, *p* = 0.014), and Spain (OR = 1.22, *p* = 0.012) but not in South Korea (OR = 1.08, *p* = 0.591). The sense of mastery was found to be a protective factor against cyberharassment victimization in the U.S. (OR = 0.80, *p* = 0.006), Finland (OR = 0.83, *p* = 0.017), and South Korea (OR = 0.68, *p* = 0.015), but not in Spain (OR = 0.88, *p* = 0.152).

The country differences were analyzed with country interaction in the logistic model, including all participants (*N* = 4816). This analysis showed that social media sharing had a stronger association with cyberharassment victimization in Finland than in the U.S. (OR = 0.93, *p* = 0.030) and Spain (OR = 0.92, *p* = 0.020).

## 4. Discussion

This cross-national study introduced and applied an integrative model of cyberharassment victimization. We combined the routine activity theory (RAT) and the general theory of crime (GTC) with personal resources to analyze risk factors for cybervictimization. The role of a sense of mastery was examined as a protective personal factor. Our study shows that despite the usefulness of individual established theories of crime in explaining cyberharassment victimization, integrating two well-established criminological theories (RAT and GTC) when explaining cyberharassment victimization in multinational studies is justified. We also see that protective factors should be considered in addition to risk factors when assessing cyberharassment victimization. The integration of the theories with risk and protective factors could provide a new and more comprehensive approach to investigating and understanding cyberharassment victimization. The current study also suggests differences in the applicability of low self-control from the GTC and sense of mastery across countries when explaining online harassment.

Cyberharassment victimization is a complex lifetime event, which in earlier literature has often been approached by studying only the factors increasing the odds of becoming a cyber victim. While there are several situational and personal exposure factors, preventative factors reduce the overall risk of cyberharassment victimization, and its consequences exist. As preventative factors can also alter the impact of cyberharassment on its victims, it could be that instead of trying to remove the negative, exposing factors from adolescents’ daily life, we should pay more attention to how positive, preventative factors could be introduced and reinforced to adolescents and young adults. In our research, a sense of mastery was defined as a protective factor for cybervictimization. The results suggest that a higher sense of mastery either provides protection against cyberharassment or individuals with a high sense of mastery ignore at least some forms of cyberharassment, thus not telling parents or other guardians that they were victims of cyberharassment.

We found differences in results between the studied countries. Cyberharassment victimization prevalence in South Korea was lower than that in other studied countries. In addition, South Korean male participants reported more cyberharassment incidents than their female counterparts. These findings in South Korea are opposite to the findings observed in the other studied countries. It is possible that South Korean youths use the Internet and social media for purposes that are less likely to expose them to cybercrime, such as communicating with friends and searching for information and learning [97]. However, the reasons behind the low prevalence and difference between the number of cyberharassment cases reported by males and females in South Korea could be the focus of future research. In addition, more research needs to be performed to determine why the sense of mastery was not a significant protective factor in the Spanish sample. Further, more multinational analyses need to be performed to show these kinds of differences between countries, which are important, especially from a preventative point of view.

Individuals can have control over how vulnerable they become or are online. Sharing personal information online could lead to a situation where individuals are more visible to bullies and tormentors on the Internet seeking suitable targets. The possibility of accessing the Internet daily and specifically visiting websites known to be malicious and containing hostile, unmoderated content increases the odds of cyberharassment, especially when users intentionally or unintentionally share information about themselves. The results from all four countries support our hypothesis 1, which states that visiting potentially harmful websites and sharing content on social media increase targets’ visibility to offenders, thus leading to cybervictimization. Loneliness implicates the lack of guardianship during troubled times, meaning that once adolescents find themselves to be cyberharassed, they perceive not to have anyone to help or protect them from the ill-doers of the Internet [45,54]. This finding emphasizes parents’ and guardians’ role in talking with adolescents about information sharing online, especially with strangers—what kind of information can or should be shared, including photos of oneself and others, what type of content is safe to share and what kind of information sharing can lead to problems. This does not mean disconnecting adolescents from social media services or the Internet, as it could lead adolescents also to be disconnected from important and supportive peer groups that promote their well-being [98].

Impulsivity can explain why certain people face difficulties in their lives more often than others. Impulsive behavior can influence a person’s daily routine through activities that make them more visible to offenders, resulting in higher odds of becoming a victim in both the physical and digital worlds [19,45,46,52,62,64]. The association found between impulsivity and cyberharassment victimization suggests that impulsivity plays a vivid role in cybervictimization in Western societies, but the role of impulsivity is not clear in Eastern societies. As our approach did not take into consideration all six dimensions of self-control, we recognize that further studies using all dimensions of GTC are needed to understand possible limitations of using GTC in multinational studies.

Finally, age and sex were not found to be conclusively associated with cyberharassment among the included countries. Only in the U.S. were both age and sex positively associated with cyberharassment victimization (i.e., females and older participants were more likely to experience cyberharassment). However, overall, this result could indicate that, because adolescents and young adults of both genders and all age groups use the Internet and social media services rather equally on a daily basis, their likelihood of encountering potential cyberharassment situations is similar. Thus, there is no lack of potential targets for motivated offenders on the Internet. In addition, women and men face different forms of cyberharassment [2,4]. Our research question was universal and could be the reason there were no significant differences between genders. It could also be that online harassment is becoming sex neutral, as noted in a study by Näsi and his research team [14].

The primary strength of our study is the comparative data derived from samples from four countries covering three continents. The data used allowed a comprehensive examination of the variables in a multinational context, using two traditional theories of cybercrime victimization. Our analyses are based on cross-national survey data; thus, there is a possibility of culturally different views and interpretations on what actions of others could be viewed as acts of cyberharassment. Additionally, the respondents’ willingness (or unwillingness) to report having been cyberharassed should be noted as a willingness to report being victimized online could vary between different countries and cultures. In individualistic cultures, people commonly define themselves in terms of their internal attributes, unlike in collectivist cultures, where individuals define themselves most commonly in terms of their social group memberships. Existing studies have also noticed that cultural norms may affect how individuals behave while online [99]. We also considered that our study’s cross-sectional design makes model testing possible only on a theoretical basis. Therefore, the theoretical approach we used should be tested further in longitudinal cross-national studies.

## 5. Conclusions

This cross-national study used a novel comprehensive integrative theory approach to investigate cyberharassment victimization among young individuals. By considering situational and personal risk and protective factors, we found that visiting potentially dangerous websites, sharing content on social media, and loneliness predict a higher likelihood of cyberharassment victimization in Finland, South Korea, Spain, and the United States. The sense of mastery may serve as a protective factor against victimization. These results highlight the continued need for safety guidelines that are clear and effective for youth navigating the Internet and social media. Professionals working with young populations, as well as parents, could benefit from up-to-date ways to monitor and uphold privacy settings. Taking into consideration their personal resources may be helpful in supporting and guiding youth in the online realm. The current study suggests that the integration of routine activity theory and the general theory of crime with personal resources can be applied to explain cyberharassment victimization in multinational studies.

## Figures and Tables

**Table 1:** Descriptive statistics. Categorical variables are presented as frequencies (*n*) and proportions (*%*). Continuous variables are presented as means (*M*), standard deviations (*SD*), and Cronbach’s alphas (*α*).

	United States				Finland				Spain				South Korea			
Variables	*M*	*SD*	Range	α	*M*	*SD*	Range	α	*M*	*SD*	Range	α	*M*	*SD*	Range	α
Danger Sites	0.79	1.79	0–9	0.86	0.92	1.36	0–9	0.70	1.09	1.90	0–9	0.84	0.42	1.92	0–9	0.85
Social Media Sharing	7.36	3.62	0–14	0.76	5.25	3.08	0–14	0.76	7.60	3.44	0–14	0.75	4.73	3.36	0–14	0.72
Loneliness	5.51	1.85	3–9	0.82	5.52	1.78	3–9	0.83	5.10	1.77	3–9	0.81	5.23	1.73	3–9	0.84
Impulsivity	1.90	1.60	0–5	0.64	1.96	1.68	0–5	0.68	2.05	1.58	0–5	0.67	1.55	1.46	0–5	0.63
Mastery	17.30	3.48	7–28	0.63	17.10	3.62	7–28	0.70	17.30	3.34	7–28	0.65	18.33	3.46	7–28	0.74
Age	20.05	3.19	15–25	-	21.29	2.85	15–25	-	20.07	3.16	15–25	-	20.61	3.24	15–25	-
Cat.variables	coding	*n*	%		coding	*n*	%		coding	*n*	%		coding	*n*	%	
Cyberharassment Victim	Yes	282	23.3		Yes	250	20.8		Yes	222	18.3		Yes	78	6.5	
	No	930	76.7		No	950	79.2		No	990	81.7		No	1114	93.5	
Participants Sex	Male	604	49.83		Male	600	50.00		Male	621	51.24		Male	591	49.58	
	Female	608	50.17		Female	600	50.00		Female	591	48.76		Female	601	50.42	

**Table 2:** Zero-order correlation matrix. Dependent variable cyberharassment victimization.

	United States	Finland	Spain	South Korea
Variables	*N* = 1212	*N* = 1200	*N* = 1212	*N* = 1192
Danger Sites	0.18 **	0.09 **	0.18 **	0.22 **
Social Media Sharing	0.16 **	0.20 **	0.14 **	0.16 **
Sex	0.07 *	0.03	0.06 *	−0.03
Age	0.10 **	−0.07 *	0.07 *	−0.08 **
Loneliness	0.18 **	0.15 **	0.13 **	0.13 **
Impulsivity	0.18 **	0.13 **	0.12 **	0.08 **
Mastery	−0.16 **	−0.14 **	−0.09 **	−0.12 **

* *p* < 0.05. ** *p* < 0.01.

**Table 3:** Logistic regression country tables for cyberharassment victimization by type of routine activity, low self-control, sense of mastery, and control variables.

Harassment	United States			Finland			Spain			South Korea		
Variables	OR	*p*	AME	OR	*p*	AME	OR	*p*	AME	OR	*p*	AME
Routine Activity												
Danger Sites	1.27	<0.001	0.038 ***	1.21	0.031	0.028 *	1.27	<0.001	0.034 ***	1.42	0.001	0.019 **
Social Media Sharing	1.32	<0.001	0.045 ***	1.73	<0.001	0.082 ***	1.28	0.005	0.035 **	1.54	0.002	0.024 **
Loneliness	1.25	0.004	0.035 **	1.41	<0.001	0.051 ***	1.23	0.010	0.029 **	1.38	0.020	0.018 *
Low Self-Control												
Impulsivity	1.34	<0.001	0.047 ***	1.19	0.014	0.027 **	1.22	0.012	0.028 *	1.08	0.591	0.004
Mastery												
Sense of Mastery	0.80	0.006	−0.035 **	0.83	0.017	−0.02 8**	0.88	0.152	−0.018	0.68	0.015	−0.022 *
Control variables												
Age	1.06	0.013	0.009 *	0.97	0.278	−0.004	1.03	0.185	0.005	0.90	0.007	−0.006 **
Female sex	1.60	0.002	0.075 **	0.92	0.613	−0.013	1.51	0.010	0.057 **	0.76	0.275	−0.015
Model *n*	1212			1200			1212			1192		
LR χ²	126.20			103.46			77.21			75.53		
Nagelkerke R²	0.149			0.129			0.100			0.156		
Model significance	0.000			0.000			0.000			0.000		

* *p* < 0.05. ** *p* < 0.01. *** *p* < 0.001. AME = average marginal effects, OR = odds ratio.

## Data Availability

YouGamble 2017 Finland (http://urn.fi/urn:nbn:fi:fsd:T-FSD3399) (accessed on 15 September 2022) and YouGamble 2018 United States (https://urn.fi/urn:nbn:fi:fsd:T-FSD3591) (accessed on 15 September 2022) are publicly available in the Finnish Social Science Data Archive. Data from South Korea and Spain will be made publicly available in the Finnish Social Science Data Archive in 2022. The data are available from the corresponding author (A.O.) with a reasonable request.
